# Real-time air pollution and bipolar disorder symptoms: remote-monitored cross-sectional study

**DOI:** 10.1192/bjo.2023.77

**Published:** 2023-06-14

**Authors:** Aaron Kandola, Joseph F. Hayes

**Affiliations:** MRC Unit of Lifelong Health and Ageing, University College London, UK; Division of Psychiatry, University College London, UK

**Keywords:** Epidemiology, rating scales, depressive disorders, bipolar affective disorders, ecological momentary assessment

## Abstract

Air pollution is associated with unipolar depression and other mental health problems. We assessed the real-time association between localised mean air quality index and the severity of depression and mania symptoms in people with bipolar disorder. We found that as air quality worsened, symptoms of depression increased. We found no association between air quality and mania symptoms.

A range of air pollutants have been found to be associated with unipolar depression in the long and short term, including particulate matter (PM), nitrogen dioxide, sulphur dioxide, ozone and carbon monoxide.^1^ However, we are aware of only one study that reports an association with symptom severity in bipolar disorder.^2^ That cross-sectional study found that increased exposure to particulate matter decreased manic episode severity, but increased the risk of mixed episodes. This finding may suggest that air pollution has depressogenic effects in bipolar disorder as well as in unipolar depression.

The digital healthcare platform juli reports levels of these air pollutants to users as the daily air quality index (AQI).^3^ Here, we examine the cross-sectional association between 2-week mean AQI and depression symptoms as measured by the Patient Health Questionnaire-8 (PHQ-8)^4^ and mania symptoms using the Altman Self-Rating Mania Scale (ASRM)^5^ in juli users with bipolar disorder.

## Method

The digital healthcare platform juli (www.juli.co) is globally available on iOS and Android. Users of juli consent to their aggregated deidentified data being used for research at the point of sign-up (www.juli.co/privacy-hub/privacy-principles). The University College London (UCL) Research Ethics Committee gave ethical approval for this study (ID 19413/002). For this study, we used data from users who stated that a psychiatrist had previously diagnosed them with bipolar disorder. Participants were recruited from the start of December 2020 until April 2022. All participants were over the age of 18.

Our exposure was mean AQI values over the 2 weeks prior to participants completing PHQ-8 and ASRM. Within the platform individuals are presented with the daily AQI for their local area, based on their smartphone geolocation (a 5 km × 5 km geospatial grid resolution globally). The AQI is calculated from the highest values of particulate matter ≤2.5 μm (PM_2.5_) and ≤10 μm (PM_10_), nitrogen dioxide, sulphur dioxide, ozone and carbon monoxide.^3^ The AQI ranges from 0 to 500, with higher values reflecting worse pollution. For each pollutant, an AQI value of 100 generally corresponds to an ambient air concentration that equals the level of the short-term national ambient air quality standard for the protection of public health. AQI values at or below 100 are satisfactory and values over 100 are unhealthy.^3^

Our outcomes were the total PHQ-8 and ASRM scores. The PHQ-8 asks eight questions about symptoms of depression over the previous 2 weeks and is a widely used clinical screening and research tool.^4^ The ASRM is a five-item multiple choice instrument covering the previous week.^5^ We examined how the mean AQI over these 2 weeks was associated with the total scores on the PHQ-8 and ASRM in individuals with bipolar disorder using linear regression. We adjusted for potential confounders of the relationship between AQI and PHQ-8/ASRM scores. These were: participants’ age, gender and mean daily step count, the mean temperature and the mean humidity over the 14 days. As participants could complete the PHQ-8 multiple times while using juli (up to twice a week), we accounted for clustering of PHQ-8 scores within participants using robust standard errors. For any significant association between AQI and our outcome measures we calculated the E-value (minimum strength of association on the risk ratio scale)^6^ to determine the unmeasured confounding needed to nullify the effect estimate. We performed all analyses using Python v3.10 for Mac (Python Software Foundation, Delaware, USA; see www.python.org).

## Results

In total, 1423 individuals with bipolar disorder completed 2930 PHQ-8 and ASRM questionnaires. Of the entire cohort, 1142 (80.3%) were female and the median age was 26 years (interquartile range 20–35). Of the included individuals, 701 (49.3%) had received a diagnosis for >5 years and 723 (50.8%) continued to see a psychiatrist regularly. The AQI values ranged from 2 to 235 (mean 43, s.d. = 23). The PHQ-8 scores ranged from 0 to 24 (mean 12.8, s.d. = 5.9) and the ASRM scores ranged from 0 to 20 (mean 4.75, s.d. = 3.9).

In unadjusted models, AQI was associated with PHQ-8 scores (coefficient 0.011, 95% CI 0.001–0.022, *P* = 0.045). After adjusting for age, gender, mean step count, temperature and humidity, we found an association between AQI and PHQ-8 score, such that total PHQ-8 score increased by 0.011 points for every 1 point increase in AQI (95% CI 0.001–0.022, *P* = 0.038). The E-value for the point estimate was 1.04 and for the lower bound of the confidence interval it was 1.01.

**Fig. 1:**
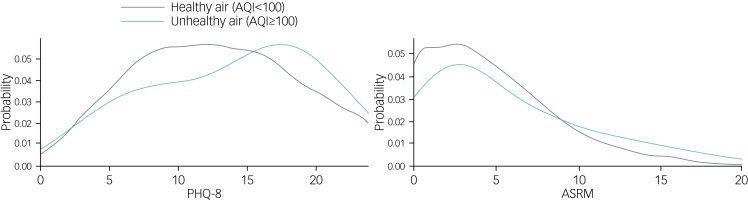
Distribution of scores on the Patient Health Questionnaire-8 (PHQ-8) and Altman Self-Rating Mania Scale (ASRM) by air quality index (AQI).

We found no association between AQI and ASRM in unadjusted (coefficient 0.002, 95% CI −0.006 to 0.010, *P* = 0.584) or adjusted (coefficient 0.001, 95% CI −0.007 to 0.009, *P* = 0.754) models. Fig. 1 shows the probability of different PHQ-8 and ASRM scores for AQI values <100 (healthy air) and ≥100 (unhealthy air).

## Discussion

Ecological studies have previously found an association between air quality and rates of bipolar disorder diagnosis.^7^ This is the first study to find that real-time air pollution exposure is associated with acutely increased depression symptoms in people with bipolar disorder. A similar association was found with short-term increases in air pollutants and unipolar depression.^1^ The effect is small, such that a one standard deviation (23 points) increase in AQI is associated with a quarter of a point increase on the PHQ-8. We did not find an association with mania symptoms, which is consistent with the results of a previous study.^2^

Our study minimised the risk of exposure misclassification that may be present in previous studies of air pollution and mental health by using participants’ mobile phone geolocation. We used a summary measure of air pollution, an approach with strengths, as most pollutants are highly correlated, but this does not allow for the study of particular types of pollution.^8^ There may be unmeasured confounders that would influence our findings, particularly the effect of socioeconomic position, area-level deprivation and urbanicity which juli does not capture. The E-value calculated for the association between a one unit increase in AQI and PHQ-8 was small (1.04), suggesting that unmeasured confounders may nullify the effect observed. Included participants may not be representative of the wider population of people with bipolar disorder as our sample were more likely to be female and to have a smart phone, and had chosen to download the juli platform. Further studies are needed to confirm this association, with measurement of additional confounders and samples that are more representative of the full population with bipolar disorder.

Air pollutants potentially play a role in mental health problems by inducing neurotoxicity, neuroinflammation and hormonal dysregulation.^9^ All of these mechanisms have been found to potentially act over short time frames.^1^ In particular, short-term exposure to PM_10_ is associated with the increased levels of cytokines (including interleukins 1-beta (IL-1β) and 6 (IL-6) and tumour necrosis factor alpha (TNF-α)) found in people with depression. Therefore, air pollution may be an important modifiable risk factor for symptom severity in various mental health problems, including bipolar disorder.

## Data Availability

An anonymised data-set is available from juli Health, given appropriate permissions.
